# The Interface Between Inflammatory Mediators and MicroRNAs in *Plasmodium vivax* Severe Thrombocytopenia

**DOI:** 10.3389/fcimb.2021.631333

**Published:** 2021-03-15

**Authors:** Marina L. S. Santos, Roney S. Coimbra, Tais N. Sousa, Luiz F. F. Guimarães, Matheus S. Gomes, Laurence R. Amaral, Dhelio B. Pereira, Cor J. F. Fontes, Ibrahim Hawwari, Bernardo S. Franklin, Luzia H. Carvalho

**Affiliations:** ^1^ Instituto René Rachou, Fundação Oswaldo Cruz, Belo Horizonte, Brazil; ^2^ Laboratório de Bioinformática e Análises Moleculares, Rede Multidisciplinar de Pesquisa, Ciência e Tecnologia, Universidade Federal de Uberlândia, Patos de Minas, Brazil; ^3^ Dep. Pesquisa Clínica e Medicina Translacional, Centro de Pesquisas em Medicina Tropical, Porto Velho, Brazil; ^4^ Departamento de Clínica Médica, Universidade Federal de Mato Grosso, Cuiabá, Brazil; ^5^ Medical Faculty, Institute of Innate Immunity, University of Bonn, Bonn, Germany

**Keywords:** *Plasmodium vivax*, malaria, thrombocytopenia, miRNA, cytokine, chemokine

## Abstract

Severe thrombocytopenia can be a determinant factor in the morbidity of *Plasmodium vivax*, the most widespread human malaria parasite. Although immune mechanisms may drive *P. vivax*-induced severe thrombocytopenia (PvST), the current data on the cytokine landscape in PvST is scarce and often conflicting. Here, we hypothesized that the analysis of the bidirectional circuit of inflammatory mediators and their regulatory miRNAs would lead to a better understanding of the mechanisms underlying PvST. For that, we combined Luminex proteomics, NanoString miRNA quantification, and machine learning to evaluate an extensive array of plasma mediators in uncomplicated *P. vivax* patients with different degrees of thrombocytopenia. Unsupervised clustering analysis identified a set of PvST-linked inflammatory (CXCL10, CCL4, and IL-18) and regulatory (IL-10, IL-1Ra, HGF) mediators. Among the mediators associated with PvST, IL-6 and IL-8 were critical to discriminate *P. vivax* subgroups, while CCL2 and IFN-γ from healthy controls. Supervised machine learning spotlighted IL-10 in *P. vivax*-mediated thrombocytopenia and provided evidence for a potential signaling route involving IL-8 and HGF. Finally, we identified a set of miRNAs capable of modulating these signaling pathways. In conclusion, the results place IL-10 and IL-8/HGF in the center of PvST and propose investigating these signaling pathways across the spectrum of malaria infections.

## Introduction

*Plasmodium vivax* is the most widespread human malaria parasite, placing 3.3 billion people at risk worldwide (Battle et al., 2019). More recently, the *P. vivax* burden has been aggravated by growing evidence of its presence across all regions of Africa (Twohig et al., 2019). Challenges to the control and elimination of *P. vivax* include its ability to relapse, its remarkable transmission efficiency, and low-density blood-stage infections, often undetected by routine surveillance (Price et al., 2020).

In non-immune humans, *P. vivax* causes a broad spectrum of clinical symptoms, ranging from asymptomatic parasitemia and prodromal symptoms followed by uncomplicated fever to multiple life-threatening organ failure (Bassat and Alonso, 2011). *P. vivax* has a lower pyrogenic threshold (the parasite density required to evoke a fever), and common prodromal symptoms include headache, anorexia, malaise, myalgias and/or gastrointestinal symptoms for one or more days, sometimes with periodicity (Anstey et al., 2012). It is currently a consensus that the virulence of *P. vivax* has been underestimated (Tjitra et al., 2008; Lacerda et al., 2012; Douglas et al., 2014), particularly in the presence of co-morbidities (Anstey et al., 2012). While critical gaps in the current knowledge of *P. vivax* pathophysiology exist, it is well-established that vivax malaria is associated with a robust systemic inflammatory response (Antonelli et al., 2020), occasionally more intense than in infections with its counterpart *P. falciparum* (Yeo et al., 2010; Anstey et al., 2012), in which severe malaria typically occurs. Findings suggest that tissue accumulation of *P. vivax* may occur, with the hidden biomass greatest in severe disease and capable of mediating systemic inflammation (Barber et al., 2015; Silva-Filho et al., 2020).

*Plasmodium vivax*-induced severe thrombocytopenia (PvST), characterized by platelet counts below 50,000 per mm^3^ of blood, is a common clinical complication in *P. vivax* malaria (Martinez-Salazar and Tobon-Castano, 2014; Naing and Whittaker, 2018; Punnath et al., 2019). The mechanisms leading to PvST are unclear but may be related to platelet activation, consumption and/or phagocytosis (Lacerda et al., 2011; Coelho et al., 2013; Essien and Emagha, 2014).

Growing evidence strengthens platelets as mediators of inflammation through their capacity to secrete numerous proteins upon activation or *via* their interaction with the endothelium or leukocytes (Ribeiro et al., 2019). For example, we have recently demonstrated that platelets enhance the inflammasome activity of innate immune cells (Rolfes et al., 2020). Therefore, it is possible to speculate that platelets may be critical players in *P. vivax*-mediated systemic inflammatory response.

The role of platelets in malaria is complex and multifaceted (O’Sullivan and O’Donnell, 2018). While platelets can kill circulating parasites of all major human *Plasmodium* species through the release of platelet factor 4 (PF4 or CXCL4) (Kho et al., 2018), most studies indicate a predominantly deleterious role (Gramaglia et al., 2017), which may involve the von Willebrand factor (de Mast et al., 2007), the coagulation cascade (Francischetti et al., 2008), and the protein C pathway (Turner et al., 2013). Additionally, studies involving both experimentally controlled (CHMI) (Woodford et al., 2020) or natural (Dos-Santos et al., 2020) *P. vivax* infection in humans suggest a link between platelets and endothelial activation, an essential pathogenic process in severe malaria.

Given the multifactorial mechanisms through which platelets can impact *P. vivax* malaria, we investigated here crucial factors and pathways that could underlie a fingerprint of PvST. For that, we measured the plasma concentrations of cytokines, chemokines, and growth factors in a cohort of *P. vivax* patients with varying degrees of thrombocytopenia. To gain additional insights into possibly perturbed regulatory pathways in PvST, we included a group of microRNAs (miRNAs), a class of small non-coding RNAs that regulate gene expression and seem critical to regulate platelet function (Stojkovic et al., 2019). By combining these highly sensitive methods with machine learning algorithms, we provide essential insights into the interplay between inflammatory mediators, miRNAs, and *P. vivax*-induced severe thrombocytopenia.

## Patients and Methods

### Study Participants and Sample Collection

Individuals who sought care at Brazilian malaria reference healthcare facilities in endemic areas of the Amazon Region and presented *P. vivax*-positive thick blood smear were invited to participate in the study. Exclusion criteria consisted of: (i) refusal to provide written informed consent; (ii) age below 17 years; (iii) self-reported pregnancy; (iv) mixed malaria infections (PCR-based assays); and (v) any other traceable co-morbidities. Upon enrolment, we used a standardized questionnaire to record demographical, epidemiological, clinical and hematological data. Seventy-seven symptomatic uncomplicated *P. vivax* patients, with a median age of 39 years and a proportion male: female of 4.5: 1, were enrolled in the study (**Table S1**). The interquartile range of parasitaemia was 1,900 to 7,380 parasites per mm^3^, with anaemia present in 25 (32%) of *P. vivax* patients. Fifty-four (70%) patients present thrombocytopenia (i.e., platelets below 150,000), with 9 (12%) of them classified as severe thrombocytopenia (platelets below 50,000/mm^3^), with no evidence of bleeding. Peripheral blood sample (5 mL in EDTA) was collected for each individual; plasma samples were immediately obtained after blood sampling (1,500 × g for 15 min at room temperature) and stored at -80°C until use. All *P. vivax* patients were promptly treated with chloroquine (1.5 g for 3 days) plus primaquine (30-45 mg daily for 7 days) according to the guideline-recommended by the Brazilian Ministry of Health. Additionally, plasma samples from nine age-matched non-infected healthy adults from the same endemic areas were collected as described above.

The methodological aspects of this study were approved by the Ethical Committee of Research on Human Beings from the René Rachou Institute – Fiocruz Minas (protocols # 05/2008 and # 80235017.4.0000.5091), according to the Brazilian National Council of Health. The study participants were informed about the aims and procedures and agreed with voluntary participation through written informed consent. The current study was conducted according to Laboratory biosafety and biosecurity policy guidelines of the Oswaldo Cruz Foundation (FIOCRUZ, Ministry of Health, Brazil (http://www.fiocruz.br/biosseguranca/Bis/manuais/biosseg_manuais.html).

### Multiplex Determination of Inflammatory Mediators

The plasma concentrations of 45 cytokines/chemokines/growth factors were measured with the ProcartaPlex^®^ 45-Plex Human array (eBioscience, USA) using Luminex^®^ xMAP technology (MAGPIX, Thermo Fisher Scientific, USA), as recommended. Specifically, the follow mediators were evaluated: C-C Motif Chemokine Ligand 2 (CCL2 or Monocyte Chemoattractant Protein 1, MCP-1); Ligand 3 (CCL3 or Macrophage Inflammatory Protein 1 alpha, MIP-1α); Ligand 4 (CCL4 or Macrophage Inflammatory Protein 1 beta, MIP-1β); Ligand 5 (CCL5 or Regulated upon Activation Normal T cell Expressed and Secreted, RANTES); Ligand 11 (CCL11 or Eotaxin); C-X-C Motif Chemokine Ligand 1 (CXCL1 or Growth-regulated Oncogene alpha, GRO-α); Ligand 8 (CXCL8 or Interleukin 8, IL-8); Ligand 10 (CXCL10 or Interferon gamma-induced Protein 10, IP-10); Ligand 12 (CXCL12 or Stromal Cell-derived Factor 1, SDF-1α); Placental Growth Factor 1 (PIGF-1); Hepatocyte Growth Factor (HGF); Leukemia Inhibitory Factor (LIF); Stem Cell Factor (SCF); Granulocyte-Macrophage Colony-stimulating Factor (GM-CSF); Vascular Endothelial Growth Factor A (VEGF-A); Vascular Endothelial Growth Factor D (VEGF-D); Nerve Growth Factor (bNGF); Epidermal Growth Factor (EGF); Brain-derived Neurotrophic Factor (BDNF); Fibroblast Growth Factor 2 (FGF-2); Platelet-derived Growth Factor (PDGF-BB); Interleukin (IL)-1α; IL-1β; IL-2; IL-4; IL-5; IL-6; IL-7; IL-9; IL-10; IL-12p70; IL-13; IL-15; IL-17A; IL-18; IL-21; IL-22; IL-23; IL-27; IL-31; Interleukin 1 Receptor Antagonist (IL-1Ra); Interferon alpha (IFN-α); Interferon gamma (IFN-γ); Tumor Necrosis Factor alpha (TNF-α); Tumor Necrosis Factor beta (TNF-β).

### Plasma RNA Extraction and Confirmation by Real Time PCR

Circulating and exosomal RNA were purified from 400 μL of plasma using Plasma/Serum Circulating and Exosomal RNA Purification kit (Norgen Biotek Corp., Canada), according to manufacturer instructions. In order to control extraction efficiency, a panel comprised of 5 spike-in probes (ath-miR-159a, cel-miR-248, cel-miR-254, osa-miR-414, osa-miR-442; IDT Technologies, USA) was added after second lysis buffer incubation (at concentration of 200 pM each). According to the NanoString protocol, spike-in oligos correlate to the ligation control A, which monitors ligation efficiency, independent of the miRNAs in the sample. Based on the plots for spike-in oligos versus the ligation control A, we have selected the cel-miR-254 and cel-miR-248 for normalization (**Figure S1**). After purification, RNA portion was concentrated using RNA Clean-Up and Concentration kit (Norgen Biotek Corp., Canada), following manufacturer instructions. As a control of the RNA extraction, samples were amplified for the U6 (RNU6−1) snRNA by using TaqMan^®^ MicroRNA Reverse Transcription kit, followed by qPCR amplification using QuantStudio 6 Flex (Applied Biosystems, USA), according to fabricant’s protocol. The U6 snRNA was successfully detected (**Figure S2**).

### Analysis of MicroRNA (miRNA) Expression

Detection of the miRNA was performed using NanoString nCounter technology (USA), according to manufacturer’s protocol. The Human v3 miRNA CodeSet allowed multiplex assessment of 800 miRNAs by specific molecular barcodes. Raw data was analysed with NanoString nSolver 4.0, using default quality control standards. For normalization, spike-in oligos (cel-miR-248 and cel-miR-254) were used. Only targets with average raw count above 100 were considered for further analysis in Partek Genomics Suite 7.0. Principal component analysis plot was also generated with the same software with batch effects removed. Targets presenting fold change equal or above 1.25 (p<0.05) were considered differentially expressed.H

### Data Analysis

Differences in medians were tested using Mann-Whitney test or Kruskal-Wallis test, as appropriated. Correlation between variables was assessed by using either Pearson’s or Spearman’s (rank) correlation (GraphPad Prism 7, San Diego, CA, USA) at significance level of 5%. The ensemble of inflammatory mediators differentially expressed in the study population was identified with the algorithm Comparative Marker Selection (fold change ≥ 1.5, Bonferroni p ≤ 0.05) followed by the unsupervised machine learning method of hierarchical clustering (Spearman’s correlation, average linkage) using the GenePattern platform (Broad Institute, MIT, USA). The independent association between inflammatory mediator and the number of platelets was evaluated by adjusting a negative binomial (NB) regression model with stepwise backward deletion. NB regression analysis was performed using the statistical package Stata version 12 (Stata Corp., Texas, USA). Covariates were selected for inclusion in the regression models if they were associated with the outcome at the 15% level of significance according to the exploratory unadjusted analysis. Associations with p values < 0.05 were considered significant, with β coefficient in regression representing the slope (the degree of change in the outcome variable for every 1-unit of change in the predictor variable).

Decision trees were used to select the minimal set of phenotypic features that efficiently segregated groups. The J48 method, present in Weka software (Waikato Environment for Knowledge Analysis, version 3.6.11, University of Waikato, New Zealand), was used for decision tree construction. We considered 0.25 for pruning confidence (-C parameter) and 2 to minimum number of instances (-M parameter). The Leave-one-out cross-validation (LOOCV) was calculated to estimate the accuracy of the generated model. To disclose affected molecular pathways, we performed functional enrichment analysis of the miRNAs targets with experimental evidence using the Ingenuity Pathway Analysis (IPA) (Qiagen) in its default parameters. IPA was parameterized as follows: core analysis run with the cutoffs: p-value = 0.05; fold change boundaries at -1.25 and 1.25; (reference set: Ingenuity Knowledge Database (Genes + Endogenous Chemicals); networks: interaction and causal confidence: experimentally observed + high predicted. From a panel of 800 miRNAs existing in the NanoString Human v3 miRNA CodeSet, only 17 miRNAs were reliably detected in plasma samples from the studied population. Therefore, the initial pathway analysis included those 17 miRNAs. The whole miRNA NanoString dataset with the original files was deposited to GEO and can be accessed with the accession code: GSE162304.

## Results

### Cytokine/Chemokines Linked With *P. vivax*-Induced Severe Thrombocytopenia

**Figure 1:**
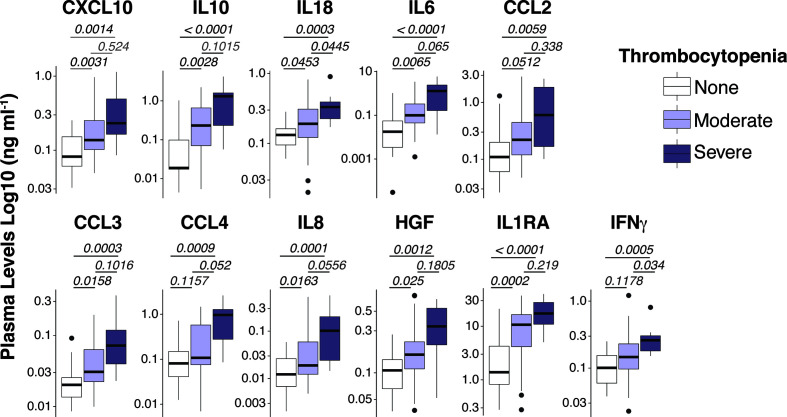
Spectrum of plasma mediators in *P. vivax* infection. Luminex Cytokine plex of cytokines, chemokines and growth factors measured in the plasma of *P. vivax* patients. Patients were stratified according to their blood platelet counts as severe (< 50,000 mm3) or moderate (50,000 – 150,000 mm3) thrombocytopenia, and non-thrombocytopenic (≥ 150,000 mm3). For each boxplot, median and interquartile ranges were represented as transversal lines in the center and lower/upper bounds of the box, respectively, with whiskers the minimum and maximum values. P values for Kruskal-Wallis multiple comparisons are shown.

To acquire a reliable assessment of the landscape of circulating cytokines/chemokines in *P. vivax* malaria, we used high throughput Luminex cytokine arrays to profile the concentrations of 45 human proteins (cytokines, chemokines, and growth factors) in the plasma of *P. vivax* patients (**Table S2**). As baseline comparison, we included plasma from nine age-matched healthy volunteers from the same localities. We found significantly altered plasma concentrations of 15 proteins in *P. vivax* patients compared to healthy donors (**Figure S3A**). Notably, several proteins in plasma from *P. vivax* patients correlated with blood platelet counts (**Figure S3B**, and **Figure 1**). Except for IL-1β and bNGF, the correlation for all other proteins was negative (**Figure S3**), suggesting an involvement of these proteins in the pathogenesis of *P. vivax* associated with severe thrombocytopenia (PvST).

**Figure 2:**
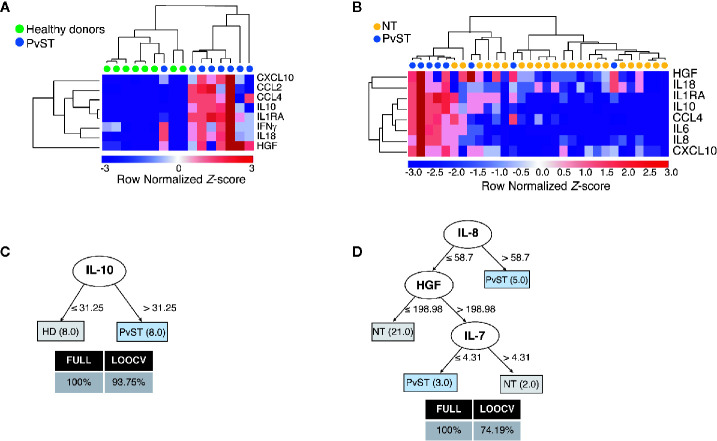
Identifying a cytokine/chemokine landscape in *P. vivax* patients with severe thrombocytopenia (PvST). **(A, B)**, Agglomerative hierarchical clustering showed key cytokines that could distinguish PvST (blue circles) from **(A)** healthy donors (green circles), or **(B)** non-thrombocytopenic patients (NT, yellow circles). Data were represented as heatmap of clustered proteins (rows) and individual plasma samples (column), with minimum and maximum normalized levels showed in blue and red scales, respectively. Hierarchical clustering was performed based on Spearman’s correlation coefficient, using the average linkage method (GenePattern, Broad Institute). **(C, D)**, Best-fit decision trees generated with the J48 algorithm (Weka software) identified IL-10 **(C)** and IL-8/HGF/IL7 **(D)** as minimum mediations that efficiently segregated PvST from healthy controls (HD) and from non-thrombocytopenic patients (NT), respectively. Weighted of the attribute (plasma levels) were placed in the root of the tree according to the cytokine/chemokine value (pg/mL) that best divided groups. The total of classified registers (correct and incorrect) for each class are given in parentheses for each terminal node with the Full training (FULL) and Leave-one-out cross-validation (LOOCV) accuracies. If incorrectly classified registers exist, they will appear after slash “/”. Data from two individuals (one control/one PvST) were not included in clustering analysis, as they have missing data (cytokine and/or miRNA with empty entries).

To gain additional insights into the relationship between circulating inflammatory mediators and PvST, we applied hierarchical clustering, an unsupervised machine learning algorithm, to investigate patterns of plasma proteins that could reliably report PvST. From all the cytokines that showed correlations with platelet counts (**Figure 1** and **Figure S3B**), this analysis highlighted eight proteins (CXCL10, CCL2, CCL4, IL-10, IL-1Ra, IFN-γ, IL-18, and HGF) as part of a specific pattern linked to PvST, as compared to unexposed healthy controls (**Figure 2A**). When we stratified *P. vivax* patients into subgroups with severe thrombocytopenia or non-thrombocytopenia, this analysis additionally identified IL-8 and IL-6, but not IFN-γ and CCL2 among the eight proteins found in the former comparison (**Figure 2B**). Using a multivariate regression analysis, we excluded the effect of possible confounding factors, such as *P. vivax* parasitic density, hemoglobin concentrations and WBC counts. Notably, the following mediators linked to PvST remained independently associated with platelet counts in *P. vivax* patients: IL-8 (β= -0.0072; p=0.006), IL-10 (β= -0.0009; p=0.005), HGF (β= -0.0026; p<0.001), and CCL2 (β= -0.0005; p= 0.006). Further, we investigate whether these arrays of mediators would allow to differentiate the subgroup of patients characterized as having moderate thrombocytopenia. The ensemble of inflammatory mediators differentially expressed in the subgroups of *P.vivax* patients (non-thrombocytopenic, moderate and severe) and healthy controls essentially selected a mixed panel of mediators abovementioned as linked to PvST (**Figure S4A**); however, hierarchical clustering analysis did not yield in a specific pattern to differentiate moderate thrombocytopenia from other groups (**Figure S4B**). Actually, the range of profile associated with moderate thrombocytopenia was not suitable to categorize this subgroup as mostly linked with up or down regulation.

Next, we employed supervised machine learning to the complete dataset to generate decision trees that could highlight key markers linked to severe thrombocytopenia from possible noise of other mediators. From all inflammatory mediators analyzed, the J48 algorithm generated best-fit decision trees that highlighted (i) IL-10 to discriminate healthy controls from PvST (**Figure 2C**) and (ii) IL-8, HGF, and IL-7 to discriminate PvST from non-thrombocytopenic patients (**Figure 2D**). Notably, inclusion of additional mediators did not result in an appreciable increase in classification accuracy. Altogether, these findings reveal a panel composed of IL-8/HGF/IL-7 and IL-10 in the center of PvST.

### A Set of miRNAs Associated With *P. vivax*-Induced Severe Thrombocytopenia

**Figure 3:**
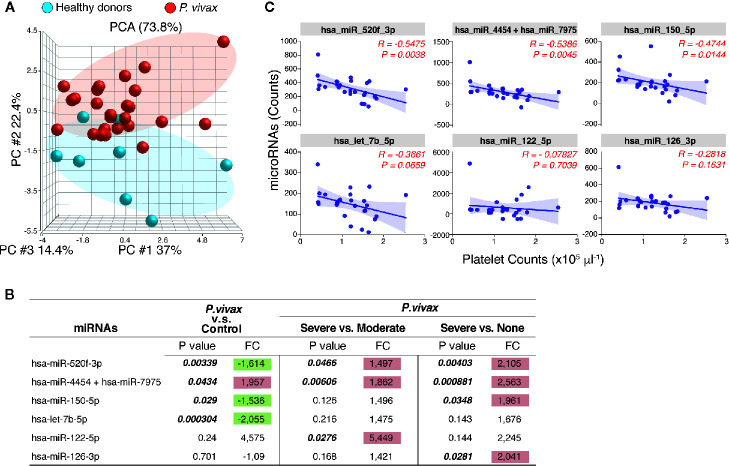
The profile of circulating miRNAs in *P. vivax* patients. **(A)**, Principal component analysis of the distribution of miRNAs detected in the plasma of a subgroup of *P. vivax* patients (red, n = 26) and healthy donors (blue, n = 8); **(B)**, Six miRNAs were differentially expressed in groups of *P. vivax* patients and healthy subjects, with positive values of fold change (FC) highlighted in red and negative values in green, considering p<0.05. P*. vivax* patients were stratified according to their blood platelet counts as: severe (< 50,000 mm3) or moderate (50,000 to 150,000 mm3) thrombocytopenia, and non-thrombocytopenic patients (≥ 150,000 mm3); **(C)**, Correlations between plasma miRNA levels and blood platelet counts in *P. vivax* patients from **(A)**. Correlation coefficients and P values are shown. For data analysis, only miRNAs with average raw counts > 100 (nCounter^®^) were considered (n=17) as follow: hsa-let-7b-5p; hsa-miR-122-5p; hsa-miR-126-3p; hsa-miR-142-3p; hsa-miR-144-3p; hsa-miR-150-5p; hsa-miR-16-5p; hsa-miR-1976; hsa-miR-199a-3p+hsa-miR-199b-3p; hsa-miR-223-3p; hsa-miR-23a-3p; hsa-miR-25-3p; hsa-miR-33a-5p; hsa-miR-4454+hsa-miR-7975; hsa-miR-451a; hsa-miR-520f-3p; hsa-miR-93-5p.

MicroRNAs (miRNAs) are well-known regulators of cytokine expression. To gain an additional layer on the mechanisms driving PvST, we used a highly sensitive molecular barcode NanoString approach to examine the miRNA profile of a representative subset of the studied population. The samples were comprised of 26 age-matched *P. vivax* patients, selected based on their blood platelet counts (as none, moderate, or severe thrombocytopenia), as well as healthy individuals, as baseline controls. Malaria patients and healthy individuals clustered into different expression ellipses in the principal component analysis (**Figure 3A**), which explained 74% of the variation in the dataset. While we detected 17 out of 800 miRNAs in *P. vivax* plasma, only six of them differed between subgroups of *P. vivax* patients or healthy controls (**Figure 3B**). Within the differentially expressed miRNA between *P. vivax* patients and controls we found that the miRNA pair hsa-miR-4454/hsa-miR-7975 was upregulated (1.957 fold, p = 0.0434) while three other miRNAs were downregulated (< 1.5-fold) in *P. vivax* patients (**Figure 3B**).

The pair hsa-miR-4454/hsa-miR-7975 was additionally significantly increased in patients with severe thrombocytopenia compared to other infections (none, or moderate thrombocytopenia) (**Figure 3B**). The miRNA hsa-miR-122-5p showed the highest expression levels in PvST patients compared to patients with moderate thrombocytopenia, but this difference was not significant when compared to non-thrombogenic patients (**Figure 3B**). Together, these findings highlight these miRNAs as associated with PvST. Supporting these findings, a correlation between the miRNA levels detected by NanoString and the blood platelet counts in these 26 patients confirmed that platelet counts are negatively associated with plasma levels of miRNAs (**Figure 3C**). Importantly, plasma miRNA levels did not show significant correlations with other blood parameters (data not shown).

In parallel, the expression of all but one miRNA linked to PvST increased alongside the plasmatic concentration of cytokines, chemokines and growth factor (**Figure S5**), which were highlighted as part of the mediators associated with PvST (**Figure 2**). Lastly, data analysis of miRNA expression confirmed that the set of miRNAs identified here are involved in key canonical pathways related to the PvST-linked mediators, including the signaling of IL-6, IL-7, IL-8/CXCL8, interferon and IL-10 (**Table S3**). To illustrate relevant networks that were identified according to the regulation predicted by the miRNA’s activities, we have included their influence on cytokine/chemokine signaling such as IL-6 (**Figure S6**), CXCL8/IL-8 (**Figure S7**), and IL-10 (**Figure S8**). Additionally, signaling pathways related to several stages of the inflammatory process and immune response were also identified, including the gene targets identified in each pathway (**Table S4**). Considering the 17 plasma-detected miRNAs, 13 of them could be experimentally validated or highly predicted by computational methods and were presented as **Supplementary Data** (**Table S5**).

**Figure 4:**
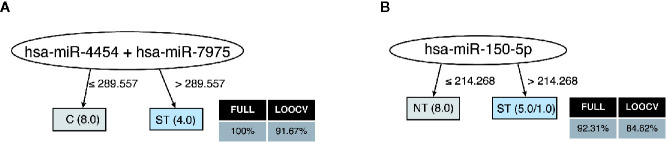
Decision trees for the miRNAs linked to *P. vivax* severe thrombocytopenia. Accurate decision trees generated with the J48 algorithm (Weka software) identified has-miR-4454/has-miR-7975 **(A)** and has-miR-150-5p **(B)** as minimum miRNA that efficiently segregated PvST (ST) from healthy controls (HC) and from non-thrombocytopenic patients (NT), respectively. Weighted of the attribute (miRNA) were placed in the roof of the tree according to the miRNA value (counts) that best divided groups. The total of classified registers (correct and incorrect) for each class are given in parentheses for each terminal node with the Full training (FULL) and Leave-one-out cross-validation (LOOCV) accuracies. If incorrectly classified registers exist, they will appear after slash “/”.

Finally, the profile of the 17 miRNAs that were detected in plasma samples of the studied subjects generated accurate decision trees based on a single miRNA for severe thrombocytopenia versus healthy controls (has-miR-4454/has-miR-7975) (**Figure 4A**), or non-thrombocytopenic *P. vivax* infections (has-miR-150-5p) (**Figure 4B**). Together, our findings provide an intricate relationship between key cytokines/chemokines with their regulatory miRNAs in *P. vivax* malaria that may be useful to define PvST.

## Discussion

Scarce, fragmented, and occasionally conflicting data are available on the cytokine/chemokine network in PvST (Park et al., 2003; Coelho et al., 2013; Raza et al., 2014; Punnath et al., 2019). We hypothesize that the combined analysis of circulating proteins and miRNAs could provide a more realistic portrait of PvST. Here, the machine learning approach’s primary purpose was to identify potential pathways that should be associated with PvST and not to use them as predictive for severe thrombocytopenia. We, therefore, examined an extensive array of soluble plasma factors in a cohort of *P. vivax* patients, whose most prominent hematological alteration was blood platelet counts, which varied from normal ranges (≥ 150 x10^3^/mm^3^) to severe thrombocytopenia (< 50x10^3^/mm^3^). As expected, no bleeding was reported in our patients, suggesting that megakaryocytes were able to release mega platelets in the circulation to compensate for the low absolute number of platelets in the periphery (Lacerda et al., 2011). In accordance, we found that the mean platelet volume (MPV), a measurement of the platelet size that increases according to platelet destruction, correlated negatively with the platelet counts (r = -0.5426, p = 0.0002).

For the detection of soluble plasma mediators, we used a Luminex-based technology that is highly reproducible compared to conventional cytokine bead arrays (Richens et al., 2010). The hierarchical clustering of samples allowed us to define two panels comprised of inflammatory and regulatory mediators that discriminated PvST from healthy controls or non-thrombocytopenic patients (**Table S6**). On the other hand, the hierarchical clustering analysis did not result in a specific pattern to differentiate moderate thrombocytopenia from other subgroups. While there is no clear explanation for this, it seems to be feasible that the great variability in the level of platelets in the group of individuals classified as having “moderate thrombocytopenia” resulted in an overlap among different *P.vivax* subgroups with a direct impact on the number of disagreements (elements in the same class but at distinct clusters and vice-versa). Consequently, the hierarchical clustering analysis may have allowed to discriminate only markedly distinct groups with non-overlapping such as PvST vs. non-thrombocytopenic (or PvSt vs. healthy controls). Notwithstanding, this interpretation may be not straightforward. For example, it has most recently been shown that that *P.vivax* patients with seemingly similar clinical presentations have distinct patterns of inflammatory response (Dos-Santos et al., 2020). These results reinforce our findings that indicate a heterogeneous host response in the setting of *P.vivax* patients present mild to moderate thrombocytopenia, a finding to be further explored. In this sense, one can claim that these inflammatory mediators may be contributing in different ways between moderate and severe thrombocytopenia and the progression is not necessarily linear in terms of mechanism. Against this, a recent hospital-based study involving a restrict number of cytokines suggested that inflammatory mediators may influence the transformation of mild forms of thrombocytopenia into severe forms (Punnath et al., 2019). Of note, this finding is also supported by our data showing that an increasing concentration of mediators linked to PvST in plasma correlated with a progressive decrease in the number of platelets in peripheral blood. Although this finding should be further explored, this reinforces the complexity of intertwined mechanisms that have previously proposed to explain malaria thrombocytopenia including peripheral destruction, bone marrow alterations, excessive removal of platelets by splenic pooling, platelet consumption by the process of disseminated intravascular coagulopathy, antibody-mediated platelet destruction and/or phagocytosis, and oxidative stress [revised in (Lacerda et al., 2011; Essien and Emagha, 2014)].

Although the same regulatory mediators (IL-10/IL-1Ra/HGF) were included in the abovementioned panels that discriminate PvST from healthy controls or non-thrombocytopenic patients, decision trees confirmed the levels of IL-10 as critical to classify PvST as compared with controls. Previous studies supported a similar association between IL-10 and decreased blood platelet counts (Park et al., 2003; Coelho et al., 2013; Raza et al., 2014; Punnath et al., 2019).High levels of IL-10 are commonly observed in *P. vivax* infections and are primarily associated with the immune system’s effort to counteract excessive inflammation (Andrade et al., 2010). This oversimplified association of IL-10 with less severe vivax disease has been challenged by studies that reported a lack of correlation between regulatory cytokines and milder symptoms. On the contrary, high levels of IL-10 are linked to intense paroxysms (Goncalves et al., 2012), increased disease-severity (Raza et al., 2013), parasite-related inflammation (Barber et al., 2015), and the occurrence of recrudescence of blood-stage infections (Chaves et al., 2016). Although previous studies highlight that IL-10 has complex and not well-characterized functions in *P. vivax* pathogenesis (Barber et al., 2015), our findings strengthen this cytokine’s critical contribution in *P. vivax*-induced thrombocytopenia.

In addition to IL-10, our analysis identified IL-1Ra as associated with *P. vivax*-severe thrombocytopenia. IL-1Ra is a naturally occurring member of the IL-1 family that binds to IL-1 receptors and antagonizes IL-1α/IL-1β (Arend et al., 1998). Notably, IL‐10 is a potent inducer of IL‐1Ra, which may represent a mechanism whereby IL‐10 exerts its anti‐inflammatory effects (Tamassia et al., 2010). Interestingly, the decision tree further indicated HGF as strongly associated with PvST, particularly to differentiate subgroups of *P. vivax* patients. HGF exerts potent anti-inflammatory effects, which seem to involve a signaling cascade leading to increased expression of IL-1Ra (Molnar et al., 2004). In agreement with the upregulation of IL-1Ra in thrombocytopenic patients, we recently demonstrated that low platelet counts in *P. vivax* malaria are associated with a progressive decrease in plasma concentrations of IL-1β (Rolfes et al., 2020). While IL-1Ra in *P. vivax* infections has been little explored, elevated IL-1Ra levels have been associated with increased disease severity in *P. falciparum* malaria-infected children (John et al., 2008). These findings suggest that an excessive anti-inflammatory response may dampen the necessary inflammatory response able to control the infection (Kumar et al., 2019).

Combined with the abovementioned regulatory mediators, the panels capable of differentiating PvST from other subgroups included well-known inflammatory mediators (**Table S6**). A range of different cell types produces these cytokines/chemokines, which account for the cascade of events that lead to leukocytes recruitment, trafficking, and amplification of inflammation and *P. vivax* pathogenesis (Barber et al., 2015; Dunst et al., 2017; Antonelli et al., 2020). Interestingly, the up-regulation of IL-6 and IL-8 was critical to discriminate subgroups of vivax patients, but not from healthy subjects. IL-6 has an established role in *P. vivax* infection, particularly as a marker of systemic inflammation leading to organ dysfunction and disease severity (Barber et al., 2015). The same authors demonstrated associations between a decreased activity of plasma ADAMTS13 (a von Willebrand factor cleaving protease), lower platelet counts, and increased concentrations of IL-6, a well-known specific inhibitor of ADAMTS13.

An unexpected finding was the potential involvement of IL-8 in PvST, further validated by the decision trees. IL-8, whose expression is induced by Toll-like receptor (TLR) and IL-1R-stimulated-NF-κB signaling, is a key mediator of neutrophil recruitment (Hoffmann et al., 2002). Though IL-8 has been largely underestimated in *P. vivax* infection, a previous study noticed impaired chemotaxis of neutrophils towards an IL-8 gradient, suggesting a possible mechanism for secondary bacterial infection during *P. vivax* malaria (Leoratti et al., 2012). On the other hand, earlier studies on *P. falciparum* have reported elevated IL-8 levels in patients suffering from severe disease (Berg et al., 2014; Mahanta et al., 2014). Although IL-8-mediated thrombocytopenia has not been investigated in malaria, a significant body of evidence suggests its involvement in platelet production, destruction, and/or activation (Broxmeyer et al., 1996; Bounameaux et al., 2007; Tsirigotis et al., 2016; Zhang et al., 2019).

While the mechanism of IL-8 induced PvST is not known, our study identified a potential route involving HGF. Notably, HGF binds to cMet (a receptor tyrosine kinase) to regulate IL-8 expression (Ito et al., 2015). HGF itself has been reported to affect the proliferation and differentiation of hemopoietic stem and progenitor cells (Zarnegar and Michalopoulos, 1995). Intriguingly, a variant beta-chain of HGF forms a molecular complex with IL-7, and this naturally occurring hybrid cytokine IL-7/HGFβ exerts a potent influence on primitive hematopoietic cells (Lai et al., 2006). In the current study, the plasma levels of circulating IL-7 (a growth factor for lymphocytes) were close to the detection limits of the assay (average 4 pg/mL), which precluded definitive conclusions about its involvement in PvST; even though the decision tree algorithm identified IL-7 to classify a small part of *P. vivax* patients. Despite that, reduced peripheral levels of IL-7 have been associated with inefficient erythropoietic responses in *P. falciparum*-induced severe anemia (Kisia et al., 2019). Unfortunately, no data related to blood platelet counts were available in the abovementioned pediatric study. In our study, anemia was not a confounding factor as (i) multivariate regression analysis confirmed that hemoglobin levels were not a confounding factor for the association between plasma concentrations of IL-8 and HGF and peripheral platelet counts (ii) the majority of enrolled patients does not present anemia. Collectively, our results suggest that a mechanism involving the upregulation of IL-8 and HGF is involved in PvST. The potential contribution of the downregulation of IL-7 should be further confirmed.

Considering that cytokines/chemokines are among the most relevant proteins whose expression is regulated by miRNAs (Garavelli et al., 2018), we further identify key regulatory miRNAs that could be involved with the inflammatory profile of PvST. Remarkably, a set of six miRNAs were differentially expressed between PvST patients and other subgroups (non-thrombocytopenic or healthy controls), with the expression levels of all but one miRNA increasing with the circulating levels of strategic mediators such as IL-6, IL-8, IL-10, and HGF. While a single miRNA (miRNA hsa-miR-122-5p) seems to be differentially expressed in patients with moderate thrombocytopenia as compared with PvST, our sample size precluded further stratification of *P. vivax* patients according to different degrees of thrombocytopenia. Consequently, we concentrate in the set of miRNAs linked to severe thrombocytopenia.

It is noteworthy that platelet-derived microparticles are transport vehicles for large numbers of miRNAs (Pordzik et al., 2018). Among the miRNAs linked to PvST, we detected platelet-related miRNAs (e.g., miR-126-3p and miR-150-5p), known to mediate platelet function and reactivity (Pordzik et al., 2018; Garcia et al., 2019). This is a relevant observation as we have previously demonstrated that platelets are major sources of circulation microvesicles in *P. vivax* malaria (Campos et al., 2010) and that thrombocytopenia strongly correlates with levels of circulating nucleic acids (Franklin et al., 2011). Lastly, the set of miRNAs identified here were involved in key canonical pathways related to PvST, including the signaling of IL-6, interferon, IL-8, and IL-10. Interestingly, the same set of miRNA identified the signaling of IL-17A (IL-17, a key component of innate and adaptive immunity) as associated with PvST.

Notwithstanding, the plasma levels of IL-17A in our *P. vivax* patients did not correlate with platelet counts. Perhaps IL-17A plays a more complex role in the cascade of events that lead to PvST. IL-17A signaling mediates the production of chemokines/cytokines such as IL-8, CCL2, and IL-6 (Milovanovic et al., 2020), which were associated with PvST in our analysis. Considering that the miRNA profile in *P. vivax* infections remains poorly explored, future studies should clarify whether the miRNAs identified here are mainly involved in the regulation of these key inflammatory mediators or play a critical role in platelet function, or both.

Our study has limitations that should be considered when interpreting the results. First, we included a relatively small number of participants, which may have underpowered some statistical analyses; Additionally, the detection of miRNA in relatively small volume of plasma samples is not an easy task. Hence, despite our robust experimental protocol, this technical limitation resulted in the reliable detection of small subset of plasma miRNAs (17 out of 800), which precluded multiple test corrections of p values. Second, the multivariate model used to control confounding variables was restricted to a small number of participants, which may have excluded cytokines that could be independently associated with platelets. Despite this limitation, key mediators such as IL-10, IL-8, and HGF were clearly identified as independently associated with platelet counts.

It is also worth mentioning that *P. vivax* malaria in Brazil is mainly an occupational infection. Because of that, our study population was predominantly male adults, which precluded us from running stratified analyses based on age or gender. Nevertheless, as our study involved non-immune patients, there was no clear reason to include a vulnerable group of children.

Finally, one single time-point sampling is unlikely to provide insights into the sequence of events from different levels of thrombocytopenia to the progression of clinical disease. While our study provides the first evaluation of the relationship between miRNAs and inflammatory mediators in PvST, the results are more associative than mechanistic and indicate novel candidate cytokines/chemokine patterns associated with severe thrombocytopenia in *P. vivax* patients. As an example, our analysis highlighted key core markers such as IL-8 and HGF as potential and unexplored signaling routes involving platelets and *P. vivax* malaria infection. Overall, our study indicates an unprecedented role of the HGF signaling in a regulatory pathway involving IL-10/IL-1Ra, and identifies a set of miRNAs capable of modulating these vital chemokine/cytokine pathways. These findings warrant further investigation in the context of thrombocytopenia across the spectrum of malaria infections. Further studies should examine the mechanistic implications of the mediators identified here. This will require animal models and larger prospective patient cohorts, including stratified analyzes based on potential influencing factors such as age and gender.

## Data Availability Statement

The complete miRNA NanoString dataset (with the original RCC files) presented in this study was deposited to GEO (https://www.ncbi.nlm.nih.gov/geo) at the accession number: GSE162304. The raw data for the Luminex was included as a **Supplementary Material (Table S2)**.

## Ethics Statement

The studies involving human participants were reviewed and approved by The Ethical Committee of Research on Human Beings from the René Rachou Institute – Fiocruz Minas (protocols # 05/2008 and # 80235017.4.0000.5091), according to the Brazilian National Council of Health. The study participants were informed about the aims and procedures and agreed with voluntary participation through written informed consent. The patients/participants provided their written informed consent to participate in this study.

## Author Contributions 

Conception and study design: LC and BF. Fieldwork and recruitment of participants: MS, CF, DP. Performance of experiments: MS and IH. Data analysis: MS, IH, TS, CF, LC, BF. Bioinformatics analysis: RC, MG, LA. The drafting of the manuscript: LC and BF. All authors contributed to the article and approved the submitted version.

## Funding

This work was supported by the European Research Council (PLAT-IL-1, 714175 to BF); the National Research Council for Scientific and Technological Development-CNPq (LC); the Research Foundation of Minas Gerais- FAPEMIG (LC); the Coordination for the Improvement of Higher Education Personnel (CAPES, Finance Code 001). BF was further supported by the Germany’s Excellence Strategy (EXC 2151 – 390873048) from the Deutsche Forschungsgemeinschaft (DFG, German Research Foundation to BF). The fellowships were sponsored by CNPq (MS, CF, TS, and LC), and by the Brazil-Germany Program of Cooperation - CNPq/CAPES/DAAD (MS).

## Conflict of Interest

The authors declare that the research was conducted in the absence of any commercial or financial relationships that could be construed as a potential conflict of interest.
