# Supplementary material for: The Interface Between Inflammatory Mediators and MicroRNAs in Plasmodium vivax Severe Thrombocytopenia
Source: Front Cell Infect Microbiol. 2021 Mar 15;11:631333. doi: 10.3389/fcimb.2021.631333 (PMC8005714; doi:10.3389/fcimb.2021.631333)
Supplement: Supplementary file 1 [file DataSheet_1.docx]

Supplementary Material

# 2.1 Supplementary Figures


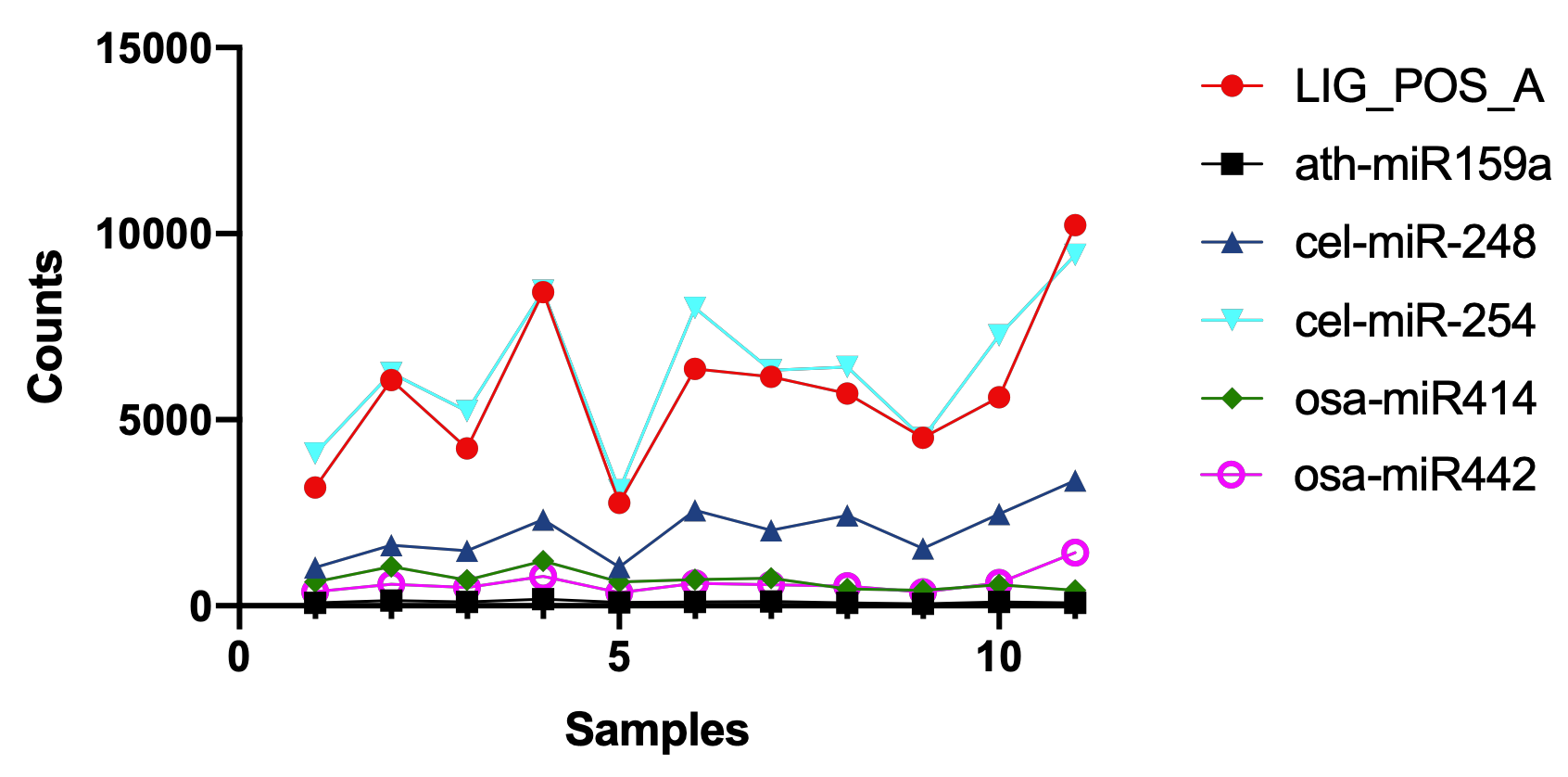


**Figure S1.** Representative NanoString nCounter assay showing the raw miRNA counts for the spike-in oligos (IDT Technologies, USA), and the ligation reporter A (Lig_POS_A) across set panel of plasma samples (n = 11). Data is from one representative of 3 independent assays.

**
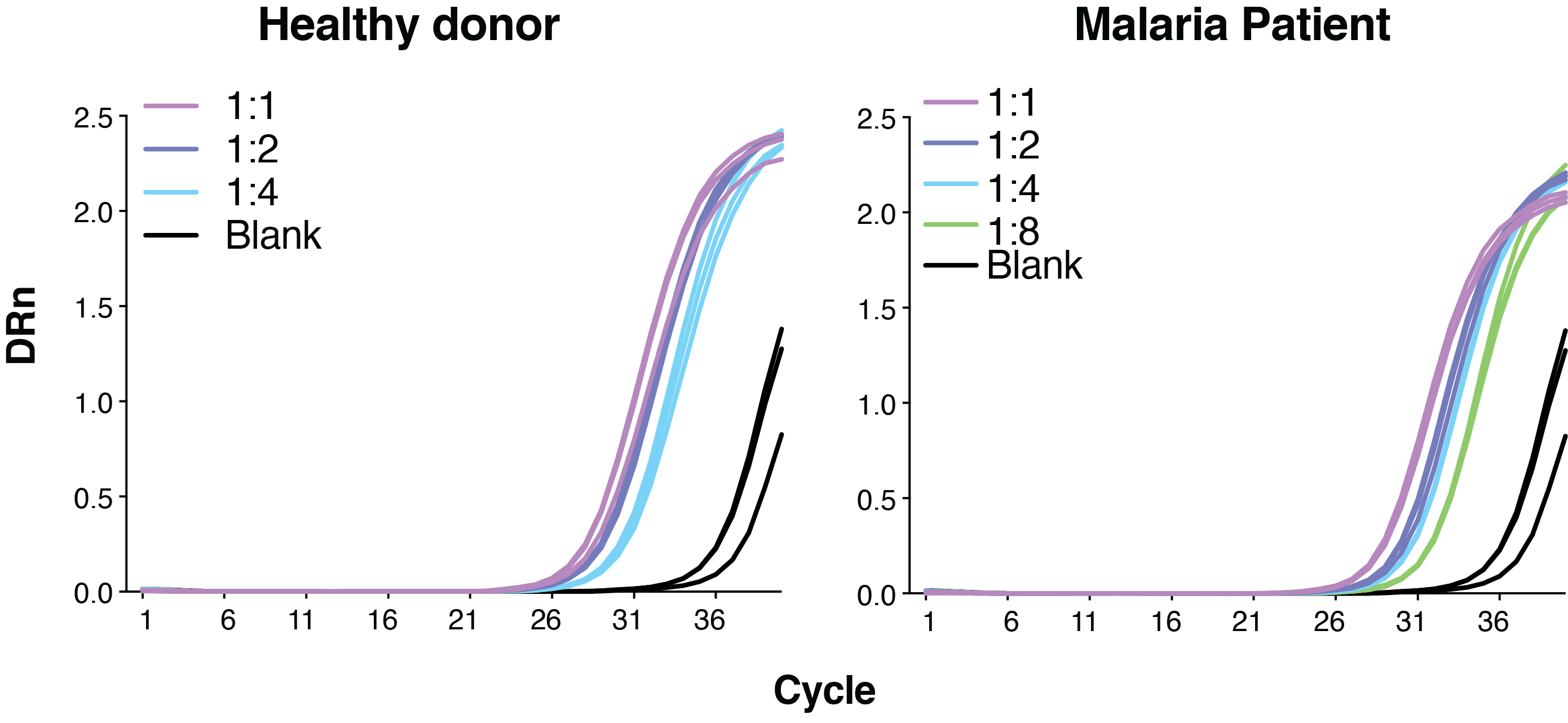
**

**Figure S2**. qPCR amplification plots for the detection of U6 snRNA in plasma from a healthy donor (left) or a patietn with *P. vivax* malaria (right). Total RNA was diluted as indicated to access detection efficiency.


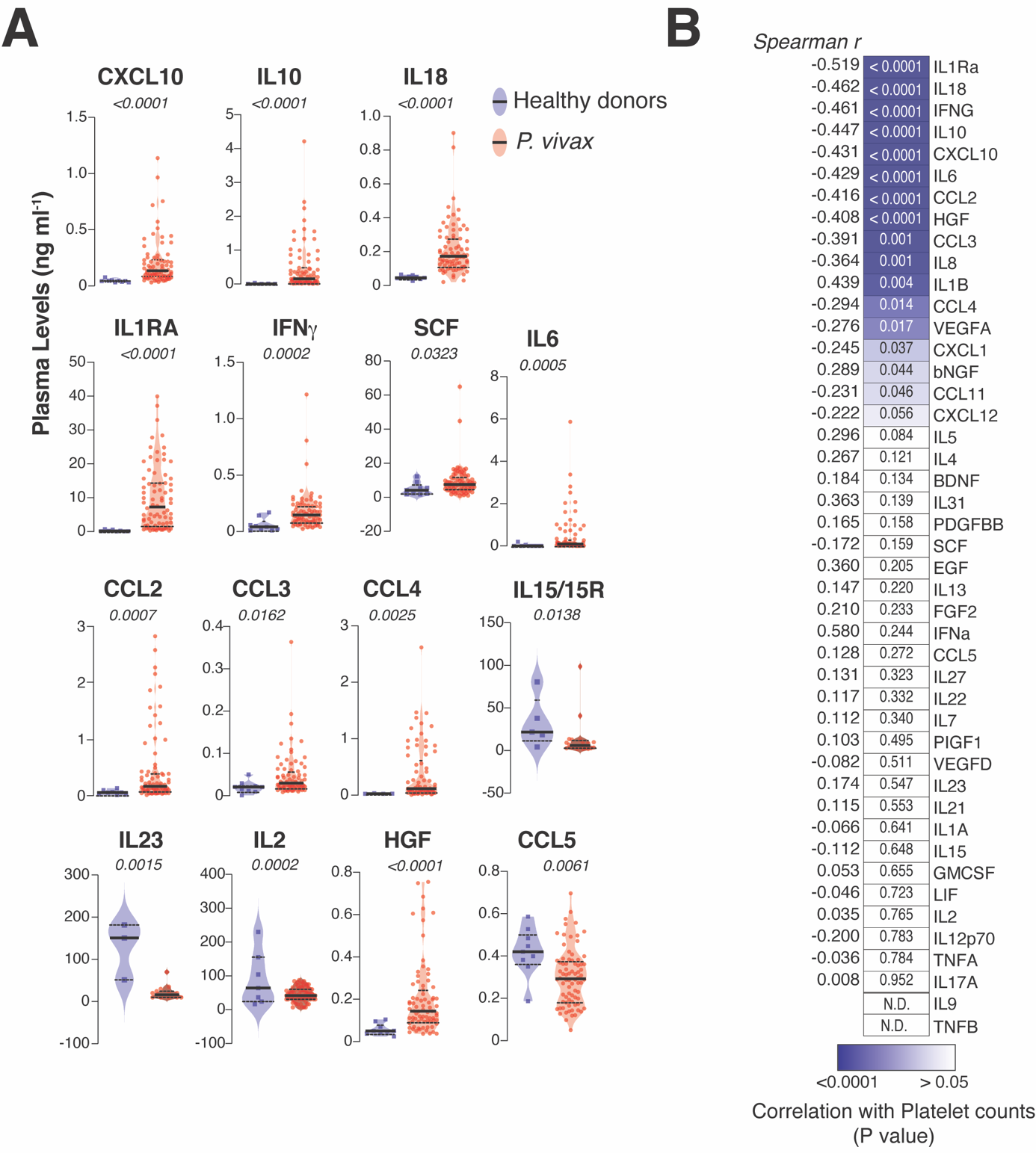


**Figure S3.** Spectrum of plasma mediators in *P. viva*x infection. **A** Luminex Cytokine plex of cytokines, chemokines and growth factors measured in the plasma of P. vivax patients or age-matched healthy donors from the same localities. Distribution shape of data are represented by violin plots with medians and interquartile ranges represented as thick and dotted lines, respectively. P values from Mann Whitney tests are shown. **B** Correlations between the plasma concentrations of 45 cytokines/chemokines and growth factors with blood platelet count in P. vivax patients. Spearman coefficient (r) and P values are indicated. IL-9 and TNF-β were not detected in plasma. Original data from IL-1β, TNF-α, and IL-18 were previously reported [20]. See Table S2 for raw data.

**
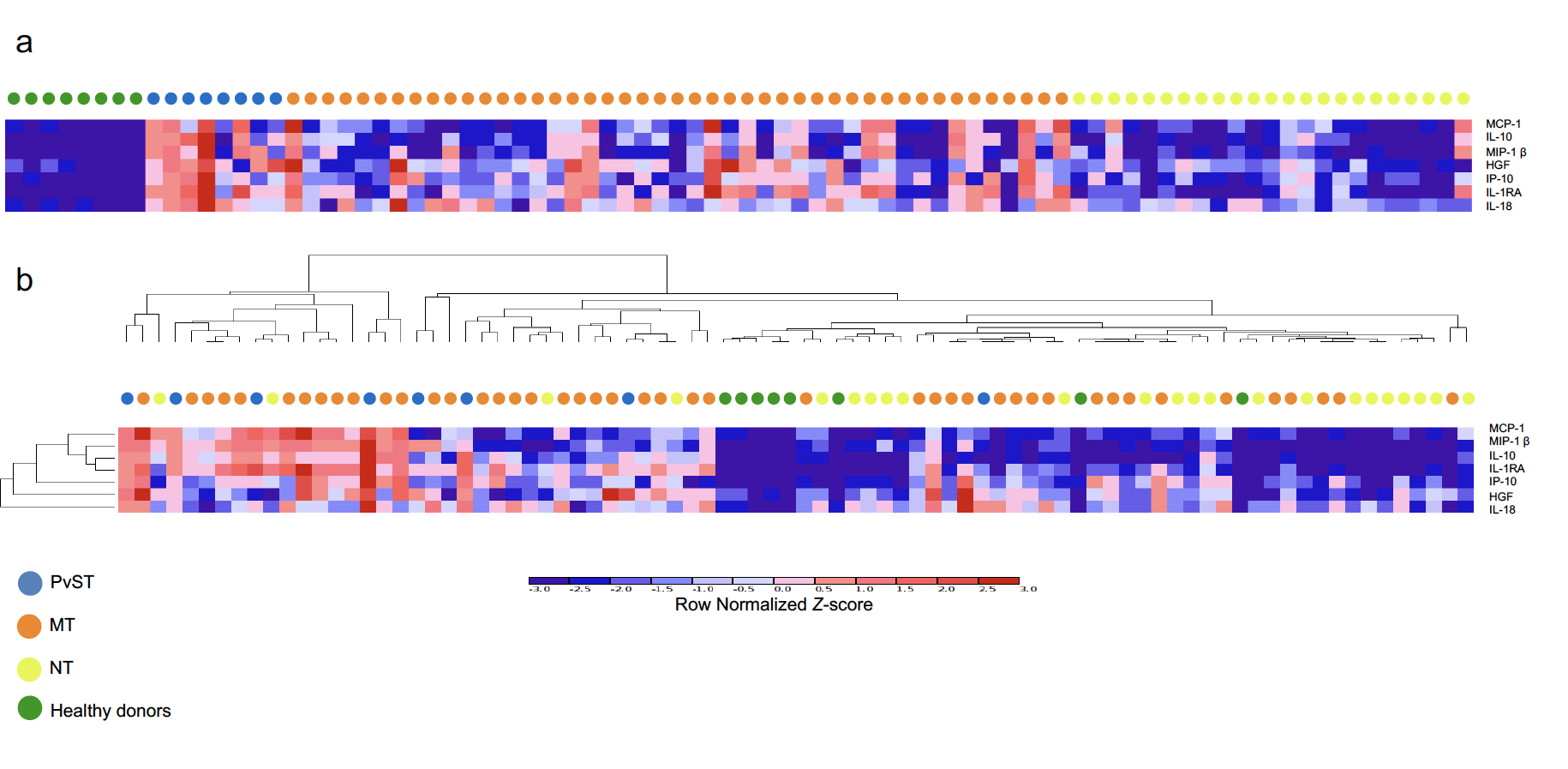
**

**A**

**B**

**Figure S4.** Cytokine/chemokine landscape for *P.vivax* patients classified as severe thrombocytopenic (PvST), moderate thrombocytopenic (MT) and non-thrombocytopenic (NT). (**A**) The ensemble of mediators differentially expressed in *P. vivax* patients and healthy donors was identified with the algorithm Comparative Marker Selection (fold change ≥ 1.5, Bonferroni p ≤ 0.05), **(B)** followed by the unsupervised machine learning method of hierarchical clustering (Spearman’s correlation, average linkage) using the GenePattern platform (Broad Institute, MIT, USA). Data were represented as heatmap of clustered proteins (rows) and individual plasma samples (column), with minimum and maximum normalized levels showed in blue and red scales, respectively.

**
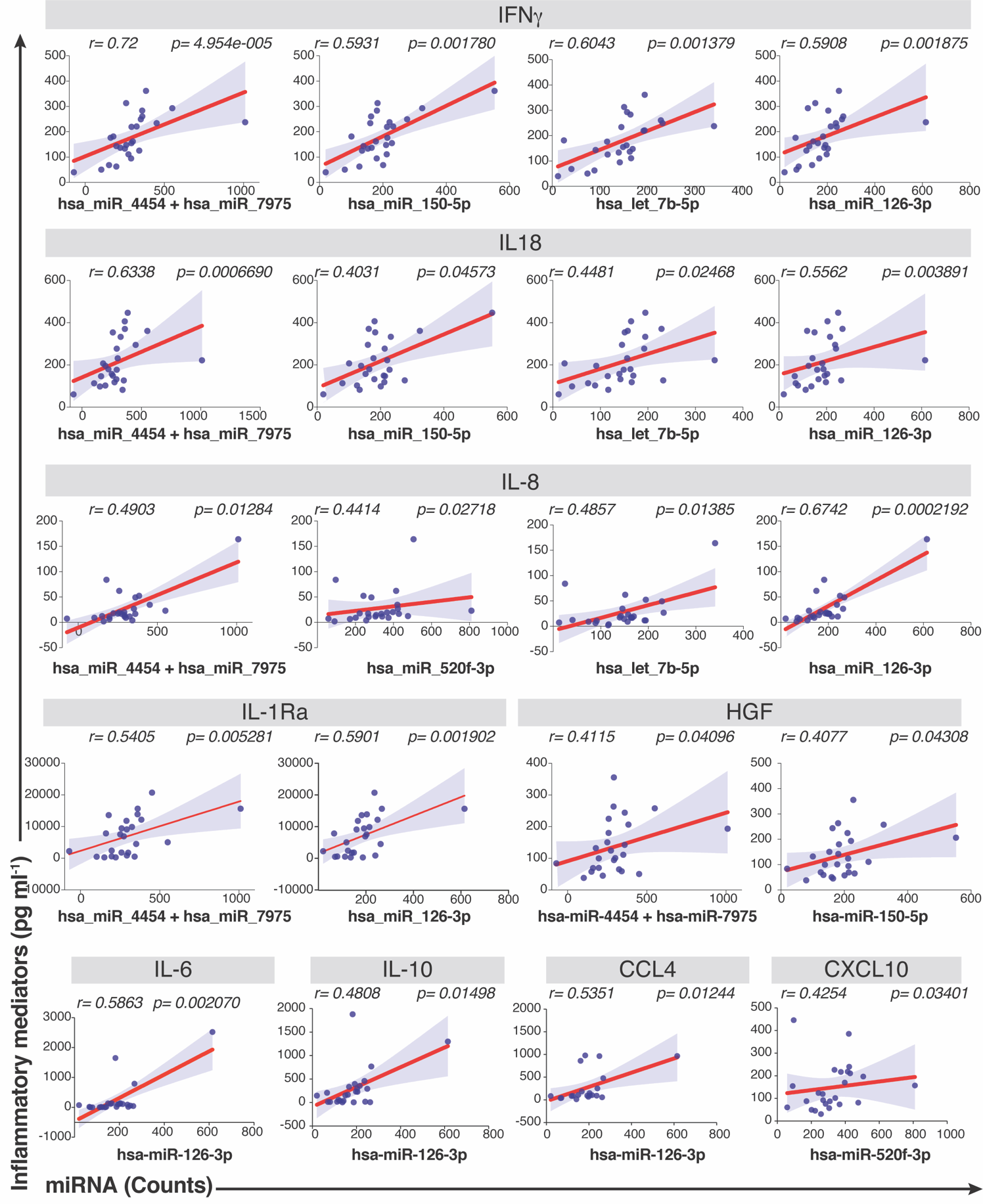
**

**Figure S5**. Correlations between plasma concentrations of miRNAs and cytokines/chemokines and growth factors linked to P. vivax thrombocytopenia. Spearman coefficient (r) and P values are indicated.

**
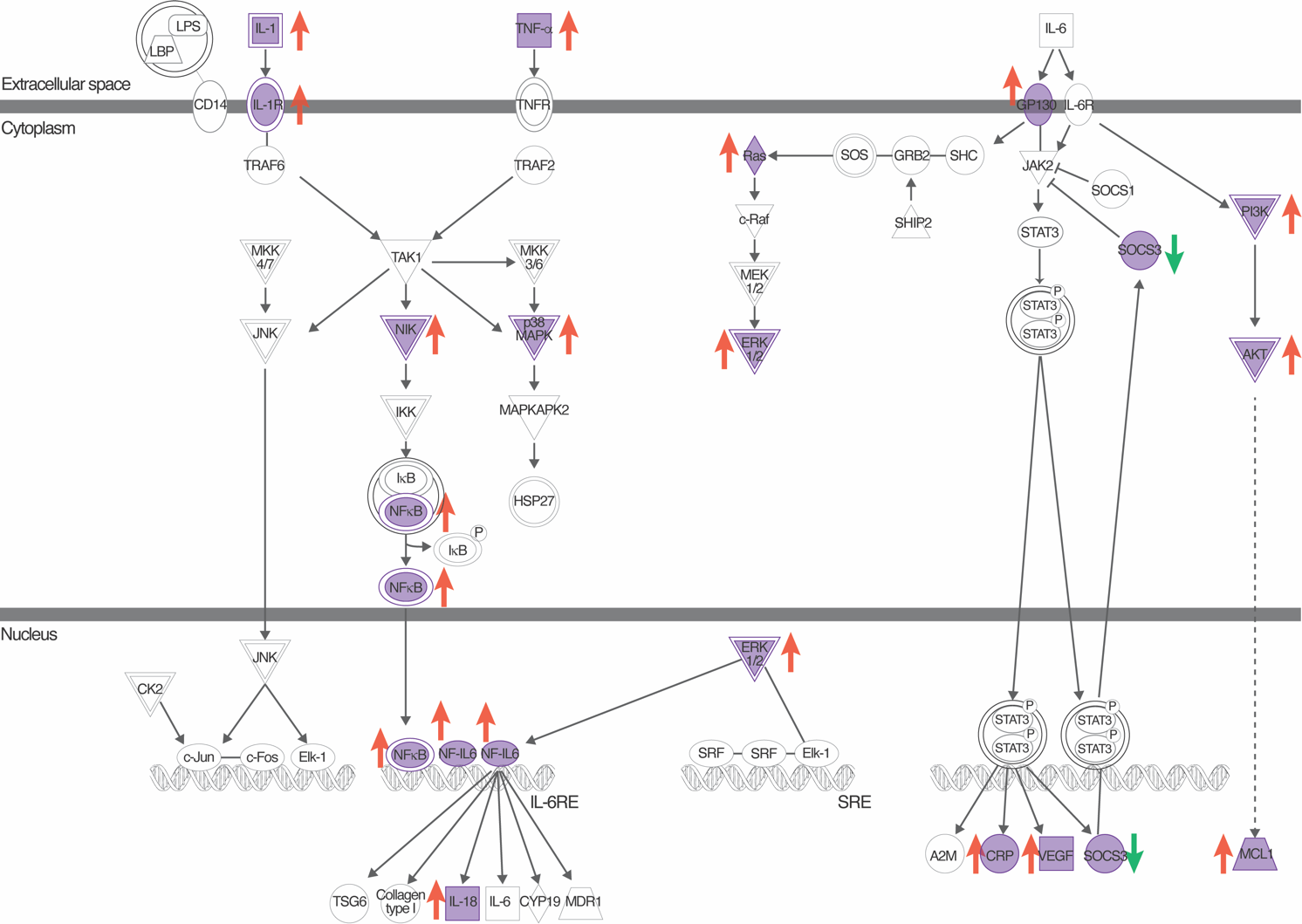
**

**Figure S6.** Schematic representation of the IL-6 signaling cascade highlighting the molecules, whose expression is influenced by the miRNAs (pink) that were differentially regulated between P. vivax patients with (ST) vs. without (NT) severe thrombocytopenia. Arrows indicate fold change (red = up, green = down). Adapted from Ingenuity Pathway, Qiagen.


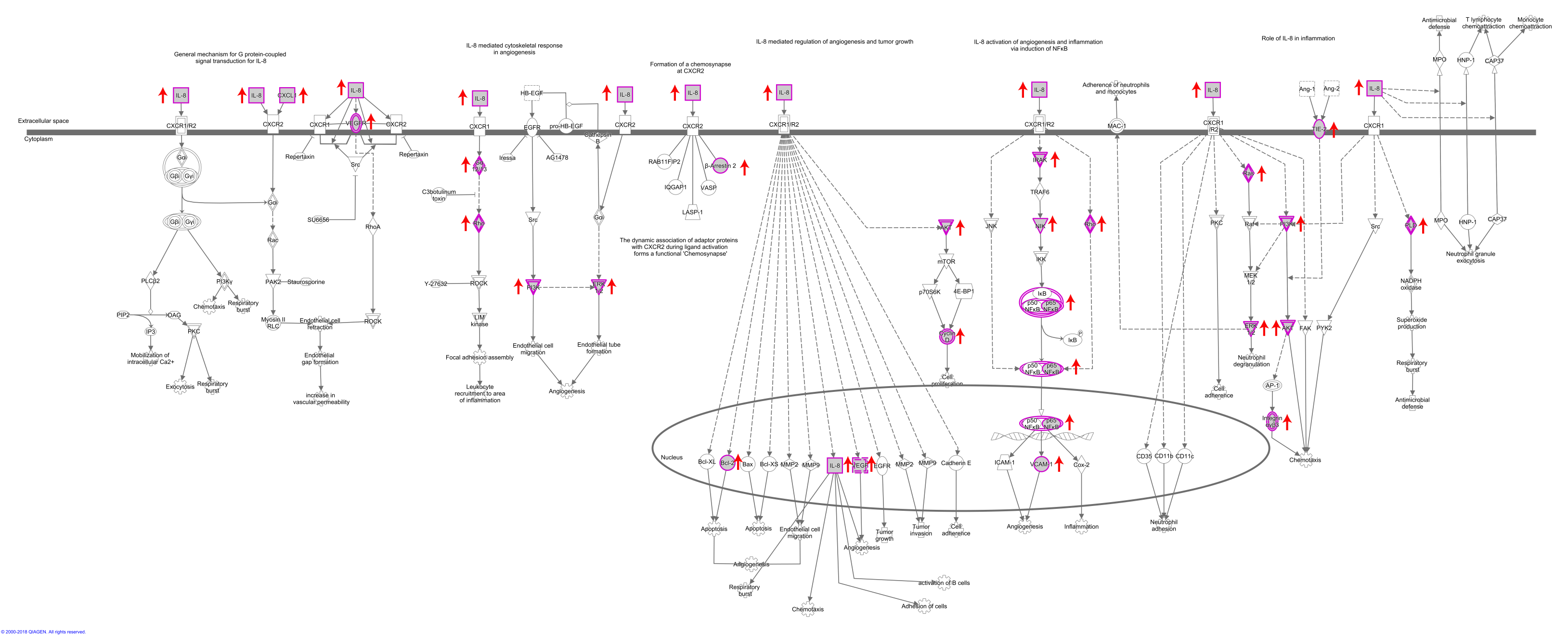


**Figure S7.** Schematic representation of the IL-8/ CXCL8 signaling cascade highlighting the molecules, whose expression is influenced by the miRNAs (pink) that were differentially regulated between *P. vivax* patients with (ST) vs. without (NT) severe thrombocytopenia. Arrows indicate fold change (red = up, green = down). Source: Ingenuity Pathway, Qiagen.


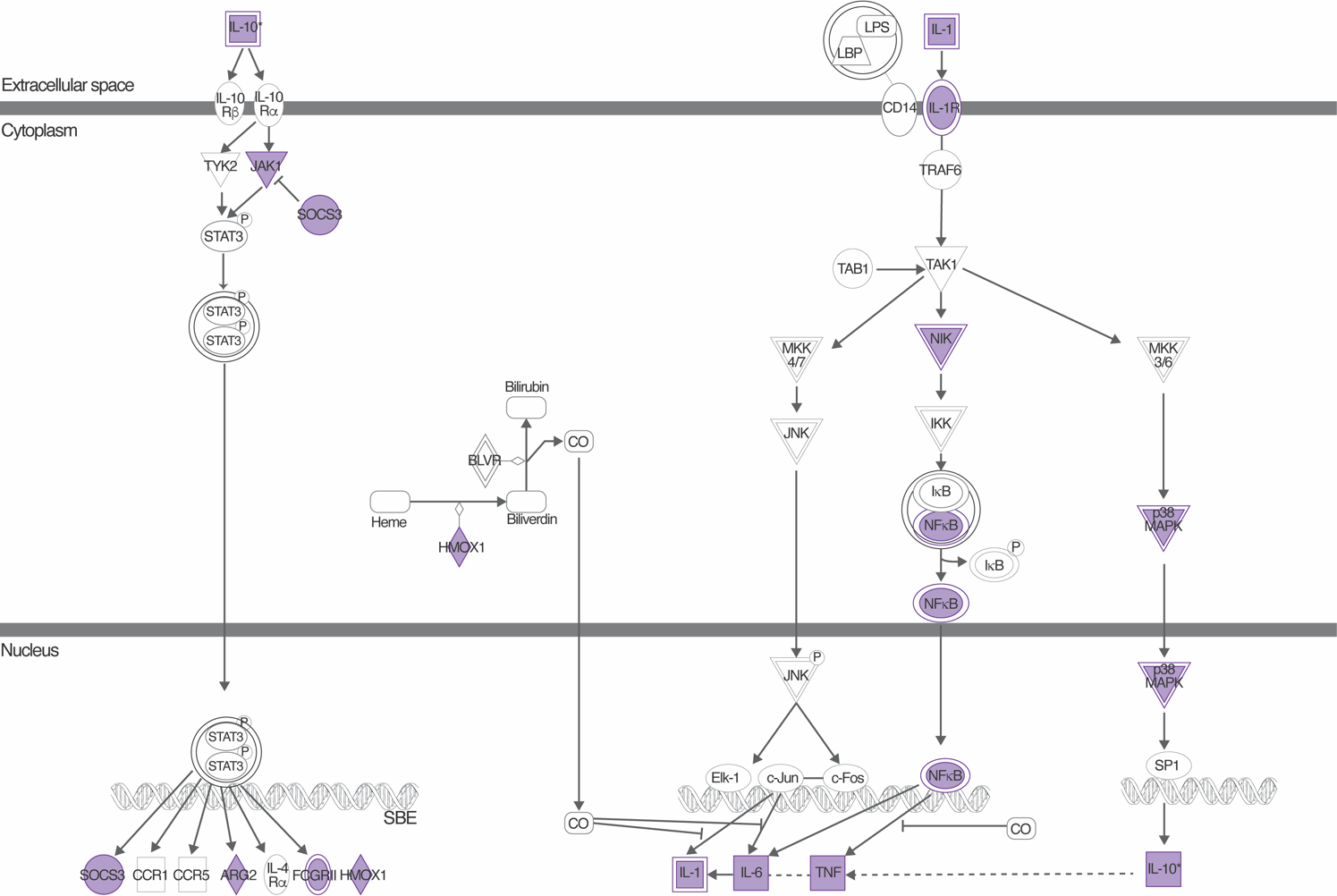


**Figure S8.** Schematic representation of the IL-10 signaling cascade highlighting the molecules, whose expression is influenced by the miRNAs (pink) and were differentially regulated between *P. vivax* patients vs. healthy volunteers. Adapted from Ingenuity Pathway, Qiagen.
