# Supplementary material for: The Interface Between Inflammatory Mediators and MicroRNAs in Plasmodium vivax Severe Thrombocytopenia
Source: Front Cell Infect Microbiol. 2021 Mar 15;11:631333. doi: 10.3389/fcimb.2021.631333 (PMC8005714; doi:10.3389/fcimb.2021.631333)
Supplement: Supplementary file 2 [file Table_1.docx]

Table S1. Demographic, epidemiological, clinical and hematological data from 77 *P. vivax* patients.

| **Parameter** | **Value** |
| --- | --- |
| Gender (Male:Female) | 4.5:1 |
| Age in years, median (IQR) | 39 (32 - 51) |
| Previous malaria episodes, median (IQR)^a^ | 3 (1 - 7) |
| Parasites per mm^3^, median (IQR) | 4,200 (1,900 - 7,380) |
| Time of symptoms in days, median (IQR) | 5 (3 - 7) |
| Leukocytes per mm^3^ (x10^3^), median (IQR) | 5.9 (4.5 - 7.3) |
| Hematocrit, %, median (IQR) | 40.3 (36.75 - 43.65) |
| Hemoglobin, g/dL, median (IQR) | 13.4 (12.5 - 14.85) |
| Anemia, n (%)^b^ | 25 (32) |
| Platelets per mm^3^ (x10^3^), median (IQR) | 103 (69 - 157.5) |
| Thrombocytopenia, n (%)^c^ | 54 (70) |
| Severe thrombocytopenia, n (%)^d^ | 9 (12) |
| IQR: interquartile range (Q1 - Q3). |  |
| ^a^Self-reported previous malaria episodes. |  |
| ^b^Hemoglobin levels below 13 g/dL and 12 g/dL for male and female, respectively. | |
| ^c^Platelet counts below 150,000/mm^3^. |  |
| ^d^Platelet counts below 50,000/mm^3^. |  |
