# Supplementary material for: The Interface Between Inflammatory Mediators and MicroRNAs in Plasmodium vivax Severe Thrombocytopenia
Source: Front Cell Infect Microbiol. 2021 Mar 15;11:631333. doi: 10.3389/fcimb.2021.631333 (PMC8005714; doi:10.3389/fcimb.2021.631333)
Supplement: Supplementary file 4 [file Table_3.docx]

Table S3. Canonical Pathways obtained from Ingenuity Pathway Analysis software.

| **Canonical Pathways** | **-log(p-value) - PvST X NT** |
| --- | --- |
| IL-17 Signaling | 3.25E00 |
| Th1 Pathway | 3.01E00 |
| mTOR Signaling | 2.9E00 |
| Neuroinflammation Signaling Pathway | 2.74E00 |
| FLT3 Signaling in Hematopoietic Progenitor Cells | 2.71E00 |
| ERK5 Signaling | 2.64E00 |
| p38 MAPK Signaling | 2.62E00 |
| Th1 and Th2 Activation Pathway | 2.41E00 |
| Apoptosis Signaling | 2.17E00 |
| IL-8 Signaling | 2.14E00 |
| Th2 Pathway | 2.09E00 |
| IL-6 Signaling | 2.03E00 |
| Interferon Signaling | 2.03E00 |
| IL-7 Signaling Pathway | 2.03E00 |
| **Canonical Pathways** | **-log(p-value) - Patients X Controls** |
| p38 MAPK Signaling | 5.17E00 |
| Neuroinflammation Signaling Pathway | 4.86E00 |
| Apoptosis Signaling | 4.55E00 |
| STAT3 Pathway | 4.38E00 |
| T Helper Cell Differentiation | 3.48E00 |
| IL-6 Signaling | 2.97E00 |
| IL-8 Signaling | 2.5E00 |
| Th1 and Th2 Activation Pathway | 2.49E00 |
| Th1 Pathway | 2.31E00 |
| IL-17 Signaling | 2.18E00 |
| Th2 Pathway | 2.18E00 |
| IL-10 Signaling | 2.16E00 |
| mTOR Signaling | 2.15E00 |
| ERK/MAPK Signaling | 2 |

PvST: severe thrombocytopenic.

NT: non-thrombocytopenic.
