# Supplementary material for: The Interface Between Inflammatory Mediators and MicroRNAs in Plasmodium vivax Severe Thrombocytopenia
Source: Front Cell Infect Microbiol. 2021 Mar 15;11:631333. doi: 10.3389/fcimb.2021.631333 (PMC8005714; doi:10.3389/fcimb.2021.631333)
Supplement: Supplementary file 7 [file Table_6.docx]

Table S6. Cytokines, chemokines and growth factors associated with *P. vivax* severe thrombocytopenia (PvST)

| **Mediator** | **Other name** | **Human gene** | **Receptor** | **Key function**^a^ | **Differential expression** | |  |
| --- | --- | --- | --- | --- | --- | --- | --- |
|  |  |  |  |  | **HC vs. PvST** | **PvST vs. NT** |  |
| **Regulatory** |  |  |  |  |  |  |  |
| IL-10 | CSIF | *IL10* | IL10R | Anti-inflammatory properties, immune response regulation | *yes* | *yes* |  |
| IL-1Ra | IL-1RN | *IL1RN* | IL1R | IL-1 inhibition | *yes* | *yes* |  |
| HGF | SF | *HGF* | Met | Cell morphogenesis regulation, inflammation regulator | *yes* | *yes* |  |
| **Inflammatory** |  |  |  |  |  |  |  |
| CCL2 | MCP1 | *CCL2* | CCR2 | Inflammatory monocyte trafficking | *yes* | ***No*** |  |
| CCL4 | MIP1β | *CCL4* | CCR5 | Macrophage and NK cell migration, T cell-DC interactions | *yes* | *yes* |  |
| IL-8 | CXCL8 | *CXCL8* | CXCR1, CXCR2 | Neutrophil trafficking | ***No*** | *yes* |  |
| CXCL10 | IP-10 | *CXCL10* | CXCR3 | T cell and NK cell trafficking | *yes* | *yes* |  |
| IFN-γ | IFG | *IFNG* | IFN-γR | Central effector of cell mediated immunity | *yes* | ***No*** |  |
| IL-18 | IGIF | *IL18* | IL-18R | Increase of natural killer cell activity, IFN-γ production | *yes* | *yes* |  |
| IL-6 | BSF2 | *IL6* | IL-6R | Inflammation, maturation of B cells | ***No*** | *yes* |  |
| ^a^Gene NCBI database; revised by [36,40,41] | | | |  |  |  |  |
| HC= Healthy controls | |  |  |  |  |  |  |
| NT= Non-thrombocytopenic *P. vivax* patients | | | |  |  |  |  |
